# Study on Preparation of Triangular Melt-Spinning Poly (Vinyl Alcohol) Fibers and Its Fabric Strengthening and Toughening Epoxy

**DOI:** 10.3390/polym13132204

**Published:** 2021-07-03

**Authors:** Ting Zhou, Meng Wang, Ning Chen

**Affiliations:** State Key Laboratory of Polymer Materials Engineering, Polymer Research Institute, Sichuan University, Chengdu 610065, China; zhouting@stu.scu.edu.cn (T.Z.); 2018223090035@stu.scu.edu.cn (M.W.)

**Keywords:** triangular PVA fibers, melt spinning, hot drawing, toughing epoxy

## Abstract

Fiber-reinforced epoxy materials have the advantages of light weight, high strength and designability, which are widely used in high-technology fields. In this paper, triangular poly (vinyl alcohol) (PVA) fibers prepared by melt spinning were used for the first time in reinforcing and toughening epoxy resins. Based on intermolecular complexation and plasticization, the triangular PVA fibers were successfully prepared via melt spinning and hot drawing. The thermal properties, crystallinity, morphology and mechanical properties of the triangular fibers with different draw ratios were characterized by DSC, FTIR, XRD, SEM and tensile testing. The results show that the comprehensive performance of the triangular fibers increased with the increase in the draw ratio. The tensile strength of triangular fibers increased from 0.3 to 4.22 cN/dtex. Then, the triangular PVA fiber and circular PVA fiber-reinforced and toughened epoxy materials were prepared, respectively. The mechanical properties of triangular PVA fiber/epoxy composites were higher than that of circular fiber-reinforced and toughened epoxy materials. Furthermore, the single-fiber pull-out test was used to analyze the interface capability of fibers and epoxy. The pull-out force of the circular fiber was 1.24 N, while that of the triangular fiber was 2.64 N. The specific surface area of the triangular PVA fiber was larger than that of the circular PVA fiber, which better made its contact with epoxy and was not easily pulled out. Experiments prove that triangular PVA fiber is an ideal material for strengthening and toughening epoxy resin.

## 1. Introduction

Over the last few years, there has been an increase in the use of fiber-reinforced epoxy resins in many areas because of their superior mechanical and physical properties, such as high specific strength and specific stiffness [1]. In fiber-reinforced composites, fibers are reinforcing materials and play a major role in the mechanical properties of the composites. The role of polymers is to bond the fibers tightly together, give full play to the mechanical properties of the fibers and transfer the external stress to the fibers [2]. 

Fibers generally used to reinforce epoxy resin are glass fibers and carbon fibers. Glass-fiber-reinforced composites have gained popularity owing to their low cost, high-strength properties, durability, ease of repair and simplicity to form [3]. D.J. Krug III et al. [4] studied the glass-fiber-reinforced epoxy–resin composites with ~50 wt.% loadings of 0°/90° cross-woven fiber glass mats for potential use as low-cost, high-strength, lightweight materials for safety/sports goggles, motorcycle helmets or window armor applications, which outperformed traditional glass-fiber-reinforced epoxy composites in properties while still providing transparency. R. Giridharan et al. [5] fabricated eco-friendly composites using glass and cotton fibers with epoxy resin by hand lay-up method. The results show that the hybrid-fiber-reinforced epoxy composites exhibited better mechanical properties than the individual samples. Furthermore, carbon-fiber-reinforced epoxy resins have attracted more attention because of their low weight, high fracture toughness, high strength and good insulating properties [6]. J.-L. Zhao et al. [7] used three-dimensional carbon fiber fabric to reinforce hydroxyapatite (HA)/epoxy composite and epoxy resin through resin transfer molding. The impact toughness and flexural strength of fiber-reinforced epoxy and fiber-reinforced HA/epoxy composites were much higher than those of epoxy and HA/epoxy composites. The impact toughness of both fiber-reinforced composites decreased while the flexural strength and the flexural modulus increased with fiber volume ratio. At present, most of the reinforced fibers used in epoxy resin are circular cross-section fibers. Compared with the circular cross-section fibers at the same denier, the profiled fibers can increase the contact area between the fiber and the matrix [8], improve the bond strength at the two-phase interface and improve fiber pull-out strength, which are proportional to the circumference of the fiber section. The profiled fiber-reinforced composite material has become a trend [9]. 

Poly (vinyl alcohol) (PVA) is a polar polymer material with multi-hydroxyl groups, which endow PVA fibers with many good properties, such as high tensile strength, excellent abrasion resistance, anti-alkaline resistance and good adhesive properties with epoxy resin [10,11,12]. Specifically, the hydroxyl group of PVA fiber can form hydrogen bonds with epoxy functional groups to enhance interface bonding. Meanwhile, profiled PVA fibers have a special surface shape, which is believed to have good effects in strengthening epoxy resin. However, the strong hydrogen bonding in PVA also makes its melting point so close to its decomposition temperature that the melt spinning of PVA is very difficult, while the solution spinning of PVA applied in the commercial field cannot form the controllable and adjustable cross-section due to the coagulation bath. In our research group, based on the principle of intermolecular complexation and plasticization, water was selected as a plasticizer of PVA. Water exists in the PVA/water system in three states: free water, freezable bound water and non-freezing water. The freezable bound water was less closely associated with PVA, while the non-freezing water directly interacted with the hydroxyl groups of PVA, retarding the water evaporation during thermal processing. Water could weaken the intra-molecular and inter-molecular hydrogen bonds of PVA molecular chains and reduce its melting point. Therefore, an environmentally friendly technique was developed to disrupt the hydrogen bonding of PVA, decreasing its crystallinity and realizing the melt spinning of PVA [13].

In this paper, based on our previous work, the triangular PVA fibers were prepared by melt spinning of PVA using water as a plasticizer, and the three-dimensional triangular PVA fabrics were first used to strengthen and toughen epoxy resin through compression molding, which provided a new type of fiber for reinforcing composites.

## 2. Experimental

### 2.1. Materials

PVA, with a polymerization degree of 1700 ± 50 and an alcoholysis degree of 99, was supplied by Sichuan Vinylon Corporation (Chongqing, China). Epoxy resin (EP), marked 128 with an epoxy equivalent of 185–192, was purchased from Chengdu Kemmett Technology Co. Ltd. From Chengdu Haihong Chemical Reagent Co. Ltd. (Chengdu, China) was purchased 4,4-diaminodiphenyl methane (DDM), which is an epoxy curing agent with an active hydrogen equivalent of 49.6. Analytically pure acetone was purchased from Chengdu Kelong Chemical Reagent Co. Ltd. (Chengdu, China) Deionized water was prepared in our laboratory.

### 2.2. Preparation of Triangular PVA Fibers Fabric

**Figure 1:**
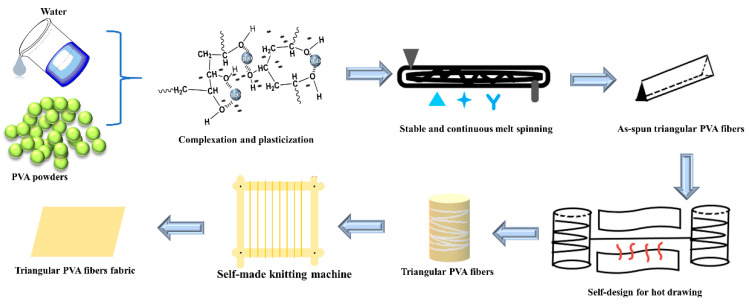
Preparation of triangular PVA fibers and fabric.

PVA pellets and deionized water were mixed and swollen in a proportion of 6:4 at about 40 °C to obtain thermoplastic PVA particles, and the thermoplastic PVA pellets were spun via a melt-spinning machine with a triangular spinneret. The extruder temperature was 80–90, 145–160 and 150–165 °C, and the spinneret temperature was 125–130 °C, rewinding with double pre-stretching to prepare as-spun triangular PVA fibers. The as-spun fibers were drawn for the second time in hot-drawing equipment; the roller temperature was 85–95 °C, the three-stage temperature of the tunnel was 180–190, 190–200 and 200–205 °C and the draw ratio was 2, 4, 6 and 7 times. Finally, the PVA fibers were woven into fabrics with a small knitting machine; the processes are shown in Figure 1. Circular PVA fibers were also prepared by the above method.

### 2.3. Preparation of PVA/EP Composites

**Figure 2:**
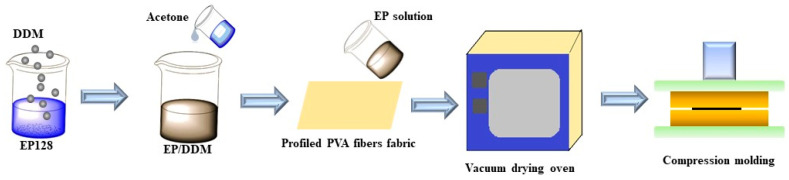
Preparation of PVA/EP composites.

Epoxy and DDM with the mass ratio of 4:1 were stirred and mixed in a 90 °C water bath. The mixture was immediately taken out and cooled to room temperature and an appropriate amount of acetone added to dilute the epoxy to obtain a 65 wt.% epoxy solution. The solution was placed into a vacuum oven for 20 min to remove air bubbles, then the solution was evenly infiltrated into the PVA fiber fabric, for which the mass ratio of fiber to epoxy resin was 1:1. The coated PVA fiber fabric was placed in a vacuum oven at 40 °C for 30 min to remove the solvent, and then hung in a fume hood for 24 h. Finally, the PVA fiber cloth was cut into the size of the mold frame of 120 mm × 120 mm, and 6 pieces of PVA fiber fabric were molded into a laminate with the procession of 80 °C for 1 h, 100 °C for 1 h and 120 °C for 2 h, as shown in Figure 2. At the curing process, the amine groups of the DDM react with the epoxy groups, creating/reinforcing the network interlock at the polymeric matrix.

As a contrast, circular cross-section PVA fibers with similar tensile strength and its fabric were used to strengthen epoxy resin through the same method as described above.

### 2.4. Characterization

The Fourier transform infrared (FT-IR) spectra of pure PVA powder and water plasticized PVA powder were obtained from a Nicolet 6700 FT-IR spectrometer (Thermo Nicolet Ltd., Vemon Hills, IL, USA).

The crystalline structure of profiled PVA fibers and as-spun PVA fibers were investigated by a DX-1000 diffractometer (Fangyuan Instrument Co. Ltd., Dandong, China), the CuKa generator system was operated at 40 kV and 25 mA, and the scanning 2*θ* ranged from 5° to 50° with a scanning rate of 0.02°/min. 

The morphology of PVA/EP composites and PVA fibers were observed by a scanning electronic microscope (SEM) (JSM-5900LV, JEOL Ltd., Tokyo, Japan) with a conductive gold layer coating and an accelerating voltage of 10 kV.

Thermal tests were performed on a TGA-Q50 (TA Instruments Co. Ltd., New Castle, DE, USA) for the thermo-gravimetric analysis (TGA) in a nitrogen atmosphere from 40 to 600 °C with a heating rate of 20 °C/min, and for differential scanning calorimeter (DSC) analysis of profiled PVA fibers by heating from 100 to 260 °C at a heating rate of 10 °C/min under nitrogen atmosphere.

The oriented structure of profiled PVA fibers and as-spun PVA fibers were investigated by a D8-discovery (Bruker. Germany) with reflection mode at a scanning time of 300 s, copper target wavelength of 0.154 nm, operating voltage of 40 kV and current of 40 mA.

Mechanical properties, including tensile strength and flexural strength of the PVA/EP composites and tensile strength of fiber, were determined at ambient temperature using an INSTRON-5567 testing machine. Each group was tested five times to take the average value. Clamp distance was 45 mm and the drawing rate was 2 mm/min. The span was 60 mm and drawing rate was 0.5 mm/min.

Impact properties of the PVA/EP composites were determined at ambient temperature using a Pit-501J plastic pendulum impact testing machine of Shenzhen Wanyan Testing Equipment Co., Ltd. (Shenzhen, China), and the pendulum size was 2 J.

## 3. Results and Discussion

### 3.1. Characterization of PVA Fibers

**Figure 3:**
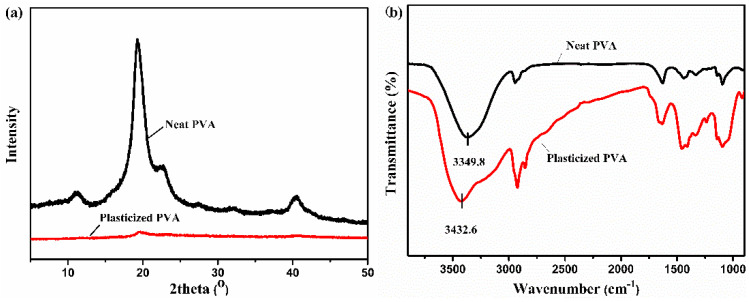
X-ray diffraction (**a**) and FTIR curves (**b**) of PVA powder and water-plasticized PVA powder.

As a result of the strong hydrogen bonding existing among PVA chains, the melting temperature of PVA was very close to its decomposition temperature, and the melt spinning of PVA was still challenged. A practical method based on intermolecular complexation was proposed to realize the thermoplastic processing of PVA [14,15,16]. Water as a strong polar molecule was introduced to realize the sufficiently stable melt spinning [17]. The plasticization is reflected in Figure 3a; the neat PVA exhibited two characteristic peaks at 2θ = 19.5° and 22.5° corresponding to the (110) and (200) reflections as the main crystalline range, respectively. Due to the excellent plasticization effect of water for PVA, the visible reduction in the main crystalline range, specifically the reduction in (110) reflection and the disappeared (200) reflection, was shown in the water-plasticized PVA [18,19]. The hydrogen bond changes between PVA molecules can be seen from Figure 3b. There were a large number of hydrogen bonds among PVA chains, which made the hydroxyl vibration peak move to a lower wavenumber at 3349.8 cm^−1^. With the addition of water, water molecules and the hydroxyl group of PVA reconstructed the hydrogen bond, destroying the hydrogen bond of the PVA molecule, causing the hydroxyl vibration peak to move to a higher wavenumber [13], located at 3432.6 cm^−1^. Based on the method of molecular complexation and plasticization, the thermal processing of PVA was realized.

**Figure 4:**
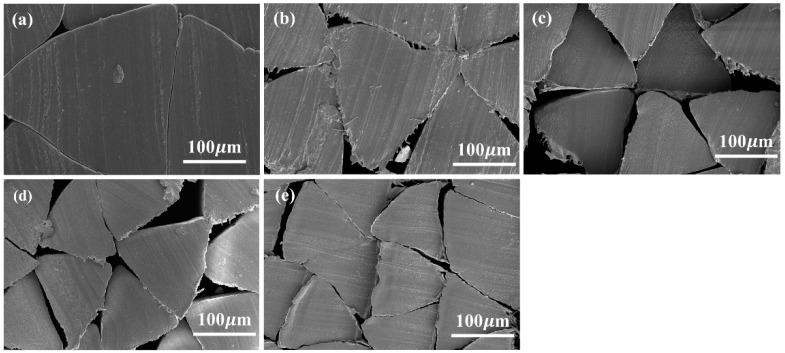
SEM images of the cross-section of triangular PVA fibers with different draw ratios: (**a**) 0; (**b**) 2; (**c**) 4; (**d**) 6; (**e**) 7.

It is well known that the cross-section is typically enlarged by the extrude swell during melt spinning, and the profiled cross-sectional shapes are changed from the original capillary shapes to circular shapes due to the surface tension of the polymer melt. The triangular PVA fibers were successfully prepared by adjusting the melt-spinning parameters, such as spinning speed and cooling speed. The cross-section morphology of triangular fiber with different draw ratios is shown in Figure 4. The as-spun fibers basically maintained the cross-sectional morphology of the original spinneret, and had a compact structure without defects such as bubbles and micropores. As the draw ratio increased, the fibers became slender, and the fiber still maintained a good triangular shape. There were no defects found in the fibers, showing that hot drawing did not cause defects in the fibers.

It is noted that Figure 4b–d has rough edges on the cross-section of fibers, because fiber bundles were fixed and then cut off in a vertical axial direction when making the sample of fiber section for SEM observation. The rough edges were caused by cutting during sample preparation, which had no great relationship with the sample itself. Compared with the equivalent circular cross-section fibers, triangular PVA fibers had a higher specific surface. Controlling the hot-drawing process, the mechanical properties of the fiber could be effectively improved while ensuring the characteristic triangular cross-section, and the high-performance triangular PVA fibers could be prepared.

**Figure 5:**
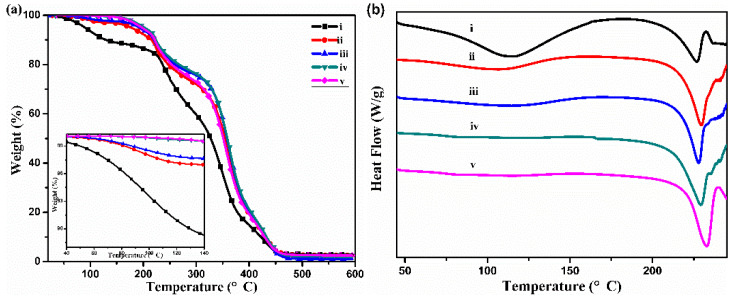
TG (**a**) and DSC curves (**b**) of triangular PVA fibers with different draw ratios: (i) 0; (ii) 2; (iii) 4; (iv) 6; (v) 7.

The TG and DSC curves of triangular PVA fibers with different draw ratios are shown in Figure 5. From the TG curves, it could be seen that the mass of as-spun fibers decreased with the increase in temperature, and the mass reduction to 90% at 130 °C was mainly the evaporation of plasticizer water. The water content of the as-spun fiber was higher than those of the stretched fibers, and with the fiber draw ratios increasing, the evaporation of water decreased obviously, because some of the water had been evaporated during the hot-drawing process. DSC curves also confirmed the above results. The enthalpy of water evaporation of the stretched fibers obviously decreased with the increase in draw ratio. The melt enthalpy of the PVA fibers increased obviously after hot-drawing, which indicated the amorphous region of profiled PVA fibers decreased and the crystalline region was more complete. 

**Figure 6:**
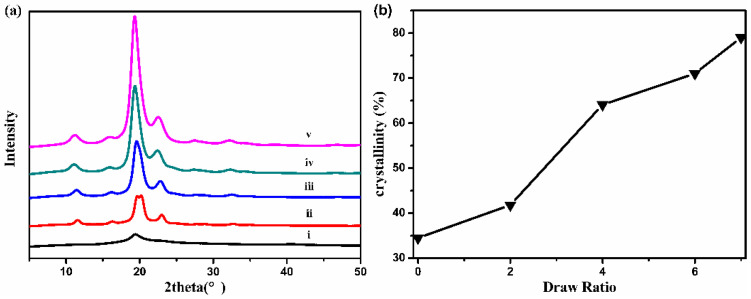
X-ray diffraction curves (**a**) of triangular PVA fibers with different draw ratios: (i) 0; (ii) 2; (iii) 4; (iv) 6; (v) 7 and the crystallinity (**b**) of triangular PVA fibers at different draw ratios.

The characteristic diffraction peak at 2θ = 19.5°(101) of the stretched fibers was obviously sharper and stronger than that of the as-spun fiber, and some new crystal diffraction peaks appeared, such as diffraction peaks at 2θ = 11.0°(100), 15.8°(001) and 22.3°(200), as shown in Figure 6a. Hot drawing made the arrangement of PVA molecular chains more regular, resulting in more perfect crystallization. The crystallinity of the fibers was quantitatively characterized by calculation. The crystallinity of as-spun triangle PVA fibers was 34.5%. The hot drawing increased the crystallinity of the fibers significantly. When it was stretched seven times, the crystallinity of the fibers was as high as 79%. During the hot-drawing process, a large amount of water evaporated, and the hydroxyl groups of PVA, originally combined with water to form hydrogen bonds, were released. The random PVA molecular chains stretched in the stretching direction during hot-drawing, and then hydrogen bonds were rebuilt, leading to the high regularity of PVA molecular chains and the crystallinity of fibers [13].

**Figure 7:**
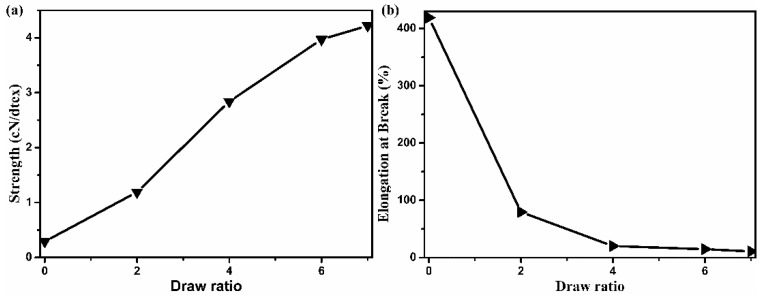
The tensile strength (**a**) and the elongation at break (**b**) of PVA triangular fibers at different draw ratios.

The orientation and crystallization of macromolecules significantly affected the mechanical properties of the fibers. The elongation at break of as-spun triangular PVA fibers was 419%, as shown in Figure 7b, indicating the potential of fibers for high ratio drawing. The as-spun PVA fibers contained more water that could form hydrogen bonds with PVA, which weakened the intra- and inter-molecular hydrogen bonds of PVA and caused PVA chains to move more easily under the action of forces, showing a larger elongation at break. After hot drawing, the tensile strength of PVA triangular fibers increased and the elongation at break decreased significantly. The tensile strength of PVA triangular fibers increased from 0.30 to 4.22 cN/dtex, and the elongation at break decreased from 419.0 to 14.7%. The improvement in the mechanical properties of PVA fibers was attributed to the hot drawing, which caused PVA chains to be arranged orderly along the stretching direction, and the crystal and orientation texture of PVA fibers tended to be perfect, thus increasing the tensile strength of PVA fibers. In this way, the PVA triangular fibers with good tensile strength were successfully prepared. 

### 3.2. Characterization of PVA/EP Composites

**Figure 8:**
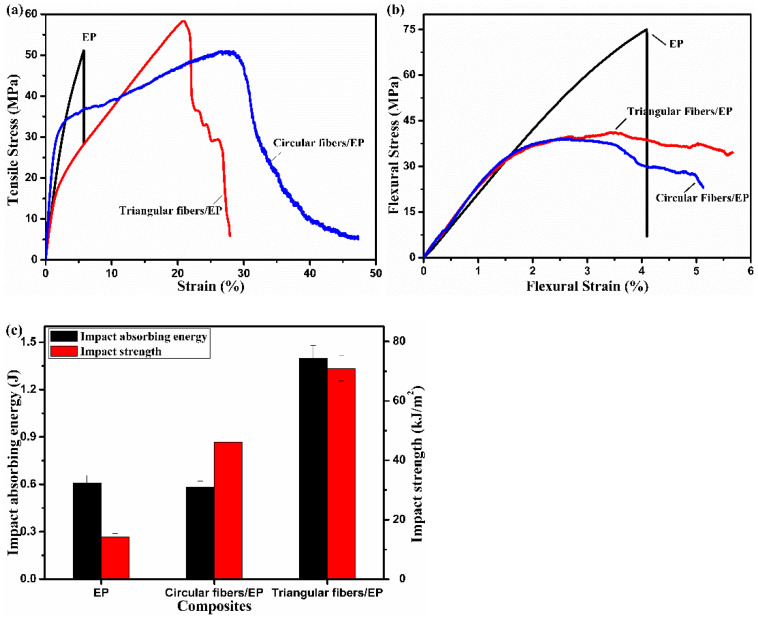
(**a**) Tensile, (**b**) flexural and (**c**) impact properties of EP and PVA/EP composites with different fibers.

Strength is the ability of a material to resist damage under external forces and a tensile test could show the strength of a material. The PVA triangular fibers with a draw ratio of seven times were used to reinforce and toughen epoxy resin. Circular PVA fibers with similar tensile strength reinforced and toughened epoxy composites and pure epoxy resin were prepared and compared with PVA triangular fibers/epoxy composites. The mechanical strength averages of composites are shown in Table 1 and the tensile stress–strain curves of EP and PVA/EP composites with different fibers are shown in Figure 8a. It can be observed from the curves that the stress of pure epoxy decreased rapidly after reaching the highest point during the stretching process, and the test bars broke directly. However, the fiber-reinforced and toughened epoxy composites had an obvious plastic deformation stage during the stretching process. The tensile strength of the composites was slightly stronger than that of the epoxy resin, while the tensile strength of the composites with circular fibers slightly lower. The elongation at break of fiber-reinforced and toughened epoxy materials with different cross-sections was greatly increased. EP resin exhibited elastic deformation while PVA fibers/EP composites exhibited plastic deformation during tensile test. The mechanical properties of composite materials were largely affected by the reinforcing phase. Compared with circular PVA fibers, triangular PVA fibers had larger specific surface area and larger contact surface with epoxy resin, causing stronger tensile strength of composites.

Stiffness is the ability of a material to resist elastic deformation under stress and a flexural test may indicate the stiffness of the material. The flexure stress–strain curves of EP and PVA/EP composites with different fibers are shown in Figure 8b. The strain at yield of PVA/EP composites with different cross-sections increased, while the flexure strength of the composites decreased, but the flexure properties of triangular PVA fibers/EP composites were better than that of circular PVA fibers/EP composites. It was worth noting that during the bending test, the composite materials bars could only be bent and could not be crushed, while the pure epoxy resin bars directly bent and broke. The flexural strength was affected by the flexural modulus and flexural strength of each component of the materials, and the compatibility between the components. The modulus difference between fiber and epoxy resin was large, so the strain generated by load was different, which could easily cause the interface damage of composites. The triangle fiber had a large specific surface area and a strong binding force with epoxy resin, so the ability of triangle PVA fibers/EP composite to resist damage was stronger than that of circular PVA fibers/EP composites.

Toughness represents the ability of a material to absorb energy during plastic deformation and fracture and an impact test shows the toughness of the material. The impact properties of EP and PVA/EP composites with different fibers are shown in Figure 8c. The cross-sections of epoxy resin bars were thicker than those of composites, so the impact absorbing energy of epoxy resin was greater than that of composites. However, the impact strength of the composites was obviously higher than that of epoxy resin, especially the triangular fibers/EP composites. Unlike pure epoxy resin, which was easy to break, the composites were not completely broken after being impacted; only the matrix was cracked and parts of the fiber layers were broken, and a small part of the fiber bundles was still connected compactly. In general, triangular fiber has great potential in strengthening and toughening epoxy due to its unique profiled structure.

**Figure 9:**
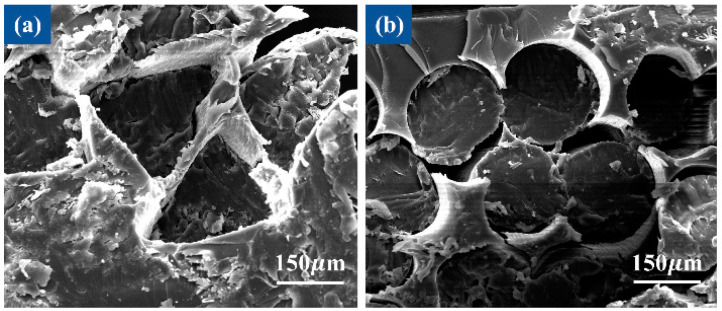
SEM images of the distribution of PVA triangular fibers (**a**) and circular fibers (**b**) in epoxy resin.

In order to clearly understand the distribution of PVA fibers in the epoxy resin matrix, the cross-section structure of the materials was observed via SEM, as shown in Figure 9. There was no obvious bubble or hole defect in the epoxy matrix, and no gap between the PVA triangular fibers and the epoxy contact surface, indicating the fibers were fully infiltrated in the epoxy solution. The fibers retained their profiled shape during the forming process. The profile structure enabled the fibers to be arranged and closely adhered. Triangular fibers were in contact with each other in a face, forming mechanical anchoring, the fiber interaction force was strong and the stress was more easily transferred between fibers when the materials were stretched. However, there was a clear gap between the PVA circular fibers and the epoxy resin matrix, the circular fibers were in line contact and the friction between the fibers was small. Compared with circular fibers, triangular fibers had a larger specific surface area, which increased the contact area with the epoxy resin matrix and increased the friction, making the fiber difficult to pull out. When the material was damaged, it was more resistant to fracture.

**Figure 10:**
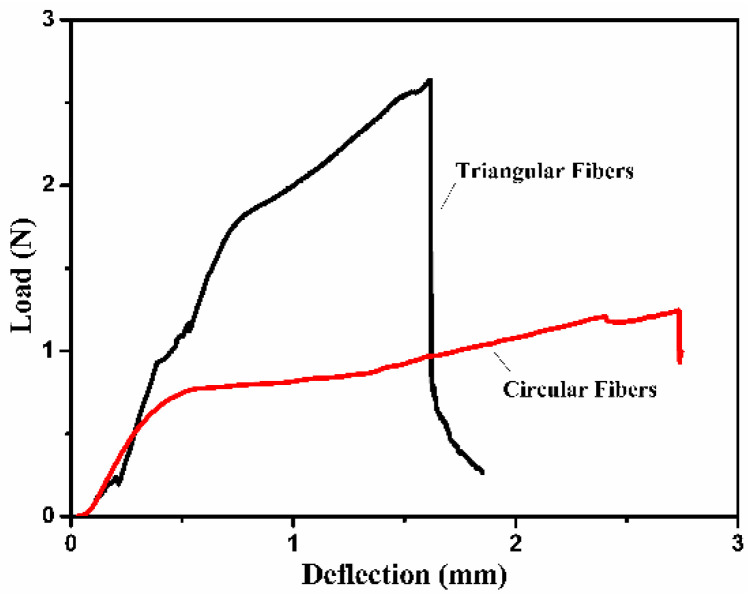
The load–deflection curves of PVA triangular fibers and circular fibers.

The mechanical properties of composite materials were affected by factors such as fibers and interfaces. Single-fiber pull-out testing (SFPOT) is an important method to evaluate the interface quality of composites. The embedded depth of the fiber in this test was 5 mm. The load–deflection curves of the triangular fibers and circular fibers are shown in Figure 10. The maximum load of the PVA triangular fibers was 2.64 N, while the maximum load of the circular PVA fibers was 1.24 N. The circular PVA fibers had a high elongation rate. When they were pulled out from the epoxy resin, the fibers deformed seriously and were easy to pull out. This was also the reason for the high elongation at break of the composite materials. Profile degree affected the specific surface area of the fibers. Triangular fibers with high specific surface area increased the contact surface with epoxy resin. In the process of pulling out, the irregular shape of the triangular fibers surface increased the friction and made the fibers difficult to pull out. On the whole, the triangular PVA fibers had good mechanical properties, which ultimately made them more effective in strengthening and toughening epoxy resin.

## 4. Conclusions

Based on inter-molecular complexation and plasticization, water was introduced as a plasticizer to weaken the intra- and inter-molecular hydrogen bonds of PVA and reduced its melting point to accomplish the melt spinning, and the PVA triangular fibers were successfully prepared via melt spinning. The hot drawing of the fibers improved the regularity of PVA chains, which made the fiber oriented and highly crystalline, so the mechanical properties of the fiber increased with the increase in the draw ratio. Triangular fibers had a higher specific surface than circular fibers which increased the contact area with the epoxy matrix, so that more force was required to pull out a single fiber. The fibers were better fixed in the EP matrix, beneficial to the improvement in the mechanical properties of the composites. The tensile performance of the triangular PVA fibers/EP composites improved, which showed obvious plastic deformation in tensile and bending tests, and the impact strength of composites was obviously enhanced, thus proving the successful preparation of the triangular PVA fiber-reinforced and toughened epoxy composites. All in all, triangular fibers had good mechanical properties and profile degree, and had a good advantage in strengthening and toughening epoxy materials.

## Figures and Tables

**Table 1:** The mechanical strength averages of EP and PVA/EP composites with different fibers.

Composites	EP	Triangular Fibers/EP	Circular Fibers/EP
Tensile strength (MPa)	51.1 ± 3.6	58.3 ± 4.2	49.6 ± 3.9
Flexural strength (MPa)	80.2 ± 5.6	35.8 ± 2.3	22.9 ± 2.0
Impact strength (kJ/m^2^)	14.2 ± 1.2	71.0 ± 4.2	46.1 ± 3.8

## Data Availability

All the data will be available to the readers.
